# Regulation of DNA Methylation by Cannabidiol and Its Implications for Psychiatry: New Insights from In Vivo and In Silico Models

**DOI:** 10.3390/genes13112165

**Published:** 2022-11-20

**Authors:** Luana B. Domingos, Nicole R. Silva, Adriano J. M. Chaves Filho, Amanda J. Sales, Anna Starnawska, Sâmia Joca

**Affiliations:** 1 Department of Biomedicine, Aarhus University, 8000 Aarhus, Denmark; 2 Translational Neuropsychiatry Unit, Department of Clinical Medicine, Aarhus University, 8200 Aarhus, Denmark; 3 Department of Pharmacology, School of Medicine of Ribeirao Preto, University of Sao Paulo, Ribeirao Preto 14049-900, SP, Brazil; 4 The Lundbeck Foundation Initiative for Integrative Psychiatric Research, iPSYCH, 8000 Aarhus, Denmark; 5 Center for Genomics and Personalized Medicine, CGPM, Center for Integrative Sequencing, iSEQ, 8000 Aarhus, Denmark

**Keywords:** cannabidiol, DNA methylation, DNMT, stress, depression, anxiety

## Abstract

Cannabidiol (CBD) is a non-psychotomimetic compound present in cannabis sativa. Many recent studies have indicated that CBD has a promising therapeutic profile for stress-related psychiatric disorders, such as anxiety, schizophrenia and depression. Such a diverse profile has been associated with its complex pharmacology, since CBD can target different neurotransmitter receptors, enzymes, transporters and ion channels. However, the precise contribution of each of those mechanisms for CBD effects is still not yet completely understood. Considering that epigenetic changes make the bridge between gene expression and environment interactions, we review and discuss herein how CBD affects one of the main epigenetic mechanisms associated with the development of stress-related psychiatric disorders: DNA methylation (DNAm). Evidence from in vivo and in silico studies indicate that CBD can regulate the activity of the enzymes responsible for DNAm, due to directly binding to the enzymes and/or by indirectly regulating their activities as a consequence of neurotransmitter-mediated signaling. The implications of this new potential pharmacological target for CBD are discussed in light of its therapeutic and neurodevelopmental effects.

## 1. Introduction

*Cannabis sativa* (cannabis) is one of the first plants cultivated by man, with historical and archaeological findings describing its cultivation in China, 4000 B.C. [1,2]. The world’s oldest pharmacopeia, the Chinese *Pen-ts’ao Ching*, described medical preparations of cannabis for treating different conditions, including pain, gastrointestinal disorders and infection, among others [1]. The first reference to the psychoactive properties of cannabis in humans is also described in this Chinese pharmacopeia, and the plant’s psychoactive effects have been well known by different civilizations since ancient times [1,3]. At the beginning of the 20th century, the medical indications of cannabis included dozens of different conditions, and its consumption for hedonistic purposes reached social importance in many countries [4]. However, concerns regarding its addictive properties resulted in worldwide legal restrictions for cannabis use for medical and recreational purposes by the second half of the 20th century [1]. Such prohibitions significantly delayed the scientific development of elucidating the plant’s chemical composition and the mechanisms responsible for the diversity of its effects.

It was only in 1964 that Gaoni and Mechoulam identified the chemical structure of the principal constituent of cannabis, Δ9-tetrahydrocannabinol (THC), which was later identified as the main one responsible for the plant’s psychostimulant effects [5,6]. Currently, it is known that cannabis contains more than 120 C21 terpenophenolic constituents named phytocannabinoids, of which THC and cannabidiol (CBD) are the most abundant [7,8]. Unlike THC, CBD does not induce psychostimulant effects nor has abuse liability [9,10,11]. On the other hand, similarly to THC, CBD is pharmacologically active and shows promising therapeutic potential in a wide range of conditions, such as chronic inflammation and pain, infection, cancer, neurological diseases and mental illnesses, among others, with varying levels of supportive evidence [9,12,13,14]. In the context of brain disorders, CBD has shown anticonvulsant, anxiolytic, antipsychotic and antidepressant effects in animal models and human studies [10,15,16,17,18]. The diverse pharmacological profile of CBD has attracted considerable attention worldwide, and many clinical trials have been designed to evaluate its therapeutic properties [19,20,21,22]. Currently, CBD is approved by the US Food and Drug Administration (FDA, Silver Spring, MD, USA) and European Medicines Agency (EMA, Amsterdam, The Netherlands) as an add-on treatment for rare epilepsies, and a preparation of 1:1 CBD+THC is approved in several European countries and Canada for the treatment of multiple-sclerosis-associated spasticity [14]. Despite some studies pointing to the beneficial effects of CBD in psychiatric disorders, the lack of sufficient supportive evidence has been an essential limitation regarding its indication in such conditions [10,23].

The growing interest of the scientific community in understanding the molecular basis of CBD effects has revealed the complex pharmacology behind its actions [14,24], making it a good candidate for further therapeutic investigation [25]. The complete elucidation of CBD’s mechanism of action in psychiatric disorders and other health conditions is thus necessary to provide a better understanding of its therapeutic potential. In this review, we address possible mechanisms by which CBD can modulate DNA methylation (DNAm), the best studied and currently the best understood epigenetic mechanism that regulates the expression of multiple genes central to the neurobiology of psychiatric disorders [26,27,28].

## 2. Cannabidiol and Its Molecular Targets

**Figure 1:**
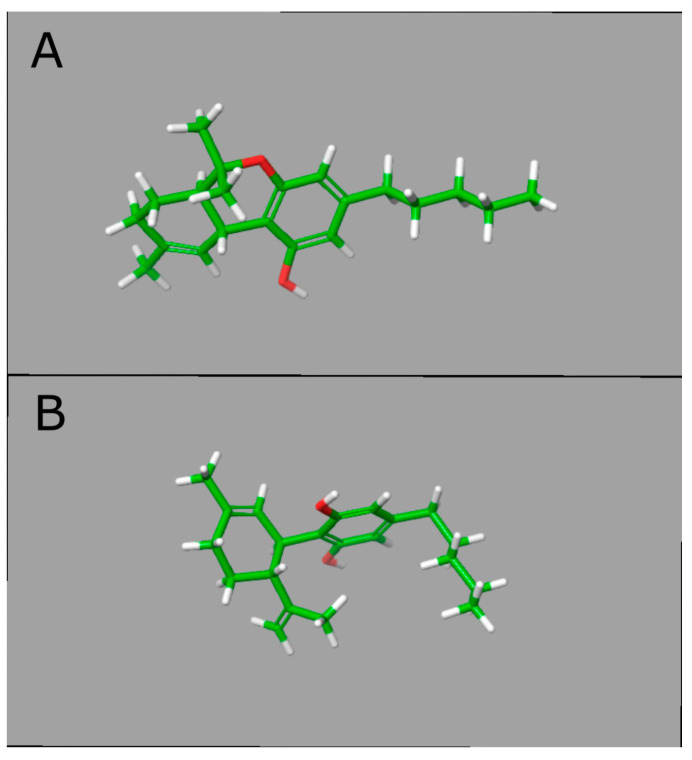
A 3D structural representation of tetrahydrocannabinol (THC) and cannabidiol (CBD). In panel (**A**) (top), the lowest energy conformation of THC is represented, and, in panel (**B**) (bottom), the lowest energy conformation of CBD. The 2D structures were retrieved from PDB with the following IDs: TCI for THC and P0T for CBD. The energy minimization and conformer generation were performed in LigPrep in Maestro-Suite v.11.2—Schrödinger. The structures were represented in Licorice model with carbon atoms represented in green, oxygens in red and hydrogens in white. Abbreviations: THC, tetrahydrocannabinol; CBD, cannabidiol; PDB, protein data bank.

**Figure 2:**
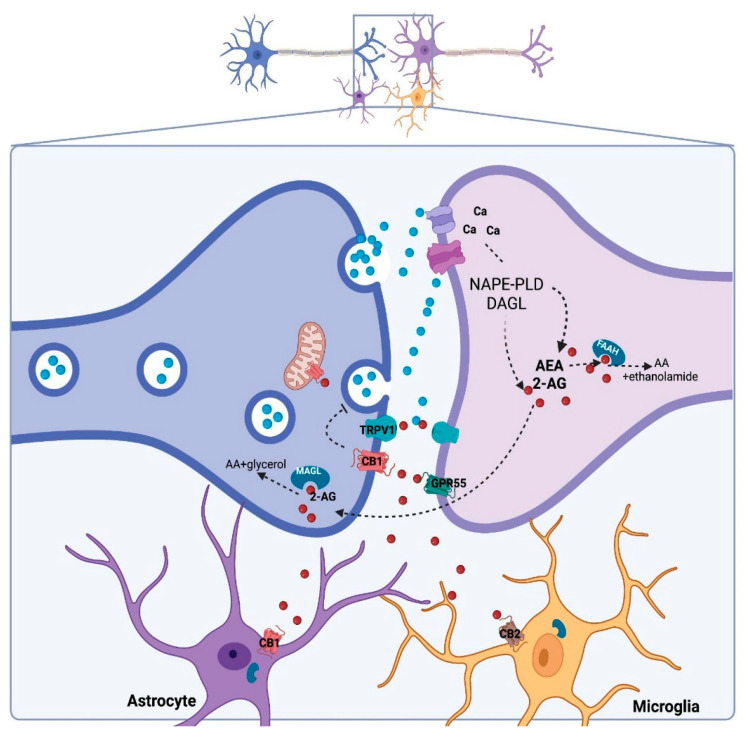
Canonical tripartite eCB signaling in the brain. Neurotransmitter-induced depolarization in the postsynaptic neuron and increased calcium concentration triggers the synthesis of anandamide (AEA) and 2-arachidonoylglycerol (2-AG) by NAPE-PLD and DAGL, respectively. The eCB can activate specific receptors (CB1, CB2, TRPV1 and GPR55) and/or follow degradation by FAAH and MAGL [31].

Although the CBD molecule is almost identical to THC, their conformational structures differ significantly, which may explain the pharmacological difference between these compounds. While THC exists in a planar conformation, CBD presents a bent structure with two rings more or less at right angles [29] (Figure 1). THC mimics the action of the endogenous cannabinoids by binding to cannabinoid receptors with much more affinity than CBD [29]. Identifying specific binding sites to THC in the brain led to the discovery of cannabinoid receptors, CB1 and CB2, culminating with the identification of endogenous cannabinoids, or endocannabinoids (eCB) [30]. The eCB system comprises the synthesizing and degrading enzymes, transporters and receptors and two main eCBs: anandamide (AEA) and 2-arachidonoylglycerol (2-AG) [16,31] (Figure 2). AEA and 2-AG are arachidonic acid derivatives and directly modulate the activation of different receptors with varying affinities and efficacies: CB1, CB2, GRP55 and TRPV1 receptors, among other receptors [16]. The concentration of eCB is tightly regulated by the degrading enzymes, the fatty acid amide hydrolase (FAAH) and monoacylglycerol lipase (MAGL), responsible for the breakdown of AEA and 2-AG, respectively [16,31]. The CB1 receptor is highly expressed in the brain and is particularly abundant in brain areas associated with motor control, emotional responses and energy homeostasis, while CB2 is primarily expressed in immune cells and glia, although it can also be present in neurons [8,16].

The CB1 receptors are the main target for THC, where it acts as an agonist, and its activation is associated with the rewarding properties of the plant [8,32]. Unlike THC, CBD has a very low affinity for CB1 and CB2 receptors [33]. An allosteric binding activity of CBD on these two receptors has been reported, resulting in CBD binding to CB1 as an inverse agonist/antagonist and CB2 as an antagonist [14,30,33,34]. In line with that, preclinical and clinical evidence indicates that CBD not only lacks abuse potential but it also attenuates the psychotomimetic and anxiogenic effects induced by THC [35,36].

Despite the negligible affinity of CBD to cannabinoid receptors, there are indirect ways in which it can activate CB1 and CB2: (a) it can inhibit FAAH, and thereby increase AEA levels [14]; (b) it can inhibit the uptake of eCBs [37]; (c) it can bind to fatty-acid-binding proteins, such as FABP5, and thereby inhibit eCB transport to FAAH [38]. Altogether, these mechanisms can increase the endogenous levels of eCB and promote activation of CB1, CB2 and TRPV1 receptors (Figure 2). Indeed, many behavioral effects of CBD can be blocked by the concomitant administration of CB1 and/or CB2 receptors [39,40,41].

**Figure 3:**
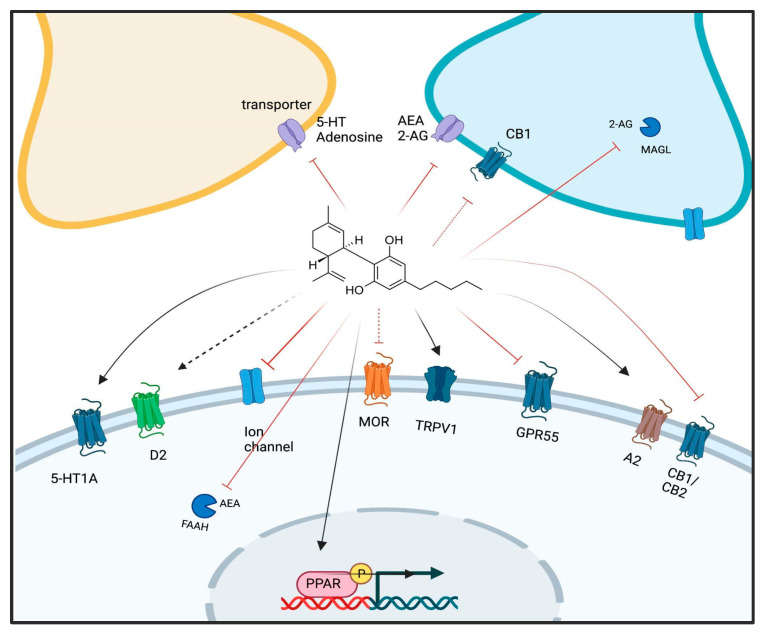
CBD molecular targets. CBD can target different molecules in the brain, including membrane (CB1, CB2, TRPV1 and GPR55) and nuclear (PPARy) receptors, enzymes and transporters, thereby regulating the neurochemical milieu and transcription in different ways [42]. Lines represent the mechanism of action: red lines = inhibitor/antagonist; dashed red lines = negative allosteric modulation; dark lines = agonist; dashed black lines = partial agonist. D2: dopamine-2 receptor; MOR: mu-opioid receptor; A2: adenosine-2 receptor.

Since CBD displays such low affinity for CB1 and CB2 receptors, many studies with CBD have attempted to characterize CB1- and CB2-independent effects for this phytocannabinoid. It is now known that CBD binds to a wide variety of targets, including receptors (primarily PPARγ, GPR55, TRPV1, 5-HT1A, 5-HT3, mu-opioid, A2 and GABA_A_), enzymes (MAGL and FAAH), transporters (monoamines, glutamate and eCB) and ion channels, among others [14,42] (Figure 3). This might explain the multitude of CBD effects on neurotransmitter release, cell signaling, gene expression and protein levels, cell cycle control, oxidative stress and inflammation [43]. Altogether, the polypharmacology of CBD may explain its ability to be effective against diverse pathologies by recruiting different mechanisms depending on the system compromised in the given condition [14,43,44]. The promiscuity of CBD on its targets also poses a challenge in deciphering its precise mechanism of action, which remains mostly unclear, especially in psychiatric disorders.

The number of studies showing the potential therapeutic effects of CBD in psychiatric disorders has considerably increased over the past decade. Overall, the reports suggest that CBD is effective against anxiety [45,46], psychotic symptoms [47,48], depression [49,50] and PTSD [51]. Furthermore, CBD is also effective in many pathological processes that are involved in the neurobiology of psychiatric disorders, such as neurodegeneration and impaired neuroplasticity [52,53], neuroinflammation [54,55], imbalanced neurotransmitter levels [56,57,58] and synaptic homeostasis [18,59]. Interestingly, these changes are under the control of transcriptional regulation involving changes in DNAm [60,61,62,63,64]. However, the number of studies for CBD effects on DNAm and its relevance for psychiatry is very limited, but the available evidence is discussed in the following sessions.

## 3. Regulation of DNAm by CBD

### 3.1. DNAm and Psychiatric Disorders: A Brief Overview

Stress exposure throughout life is amongst the most influential environmental challenges that can promote long-lasting reprogramming of gene expression and influence the development of brain disorders [63]. The epigenome translates the gene–environment interaction into gene expression changes that influence several brain processes, such as neuronal differentiation, maturation and plasticity, as well as brain neurochemistry, thereby affecting how the brain responds to challenging environmental exposures [63].

**Figure 4:**
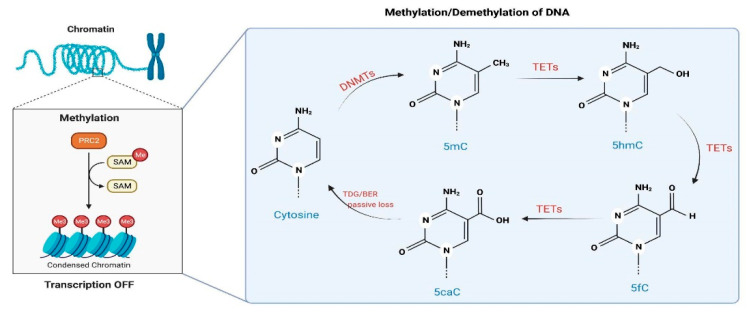
**DNA methylation (DNAm) and demethylation in mammal cells.** DNAm is catalyzed by DNMTs, which transfer a methyl radical from SAH to cytosines, forming 5mC. The methylated cytosine can become demethylated either by (i) actively undergoing a series of reactions by TETs or (ii) passively by losing the mark during DNA replication [67,68].

Amongst the epigenetic mechanisms that influence chromatin remodeling and regulation of gene expression is DNAm, characterized by the transfer of a methyl group, donated by S-adenosylmethionine, onto the C5 position of the cytosine to form 5-methylcytosine (5mC) [65]. In most mammalian cell types, DNAm occurs primarily on a CpG dinucleotide, and it is associated with repression of transcription if it happens in promoter regions, while in gene bodies, it is associated with an increase or decrease in transcription [66]. The methylation of DNA is catalyzed by a family of enzymes called DNA methyltransferases (DNMTs) (Figure 4). While DNMT1 functions during DNA replication to copy the DNAm pattern from the parental DNA strand onto the newly synthesized daughter strand, DNMT3a and DNMT3b can establish a new methylation pattern for an unmodified DNA sequence and are thus are known as de novo DNMTs [65].

Recent evidence indicates that DNAm is a dynamically regulated process with the participation of a family of enzymes named ten-eleven translocase (TET), responsible for oxidizing 5mC onto 5-hydroxymethylcytosine (5hmC), 5-formylcytosine (5fC) and 5-carboxylcytosine (5caC), in sequential reactions [69,70,71] (Figure 4). Interestingly, neuronal activation can regulate the activity of TET1, which catalyzes the conversion of 5mC to 5hmC, and induce DNA demethylation with subsequent changes in the expression of genes involved in neuronal plasticity [72]. A similar role has also been proposed for Tet3 [73]. DNAm can be an active or passive process. Passive demethylation occurs when DNAm patterns are not restored after DNA replication on the newly synthesized DNA strand, while active is thanks to the oxidation via TETs. Furthermore, 5hmC itself has different properties and impacts on gene expression than 5mC, with altered patterns of epigenetic regulation and subsequent gene expression already after the first oxidation from 5mC to 5hmC [74].

This dynamic regulation of DNAm in the brain is crucial for neuronal development and subtype specification, synaptic plasticity, neuronal activity, neuroprotection and regeneration [60,66]. Therefore, aberrations in writing or reading changes in DNAm can be linked to developing different brain disorders, including mental illnesses [26]. Detailed reviews have been published elsewhere, where aberrant patterns of DNAm have been described in peripheral and brain tissue of individuals with depression, anxiety, PTSD and schizophrenia [27,75], all psychiatric disorders with established increased risk through exposure to adverse life events [26]. Changes in DNAm (hypo/hypermethylation) have been identified in several genes coding for receptors, transporters and degrading enzymes of neurotransmitters involved in psychiatric disorder etiology and treatment (dopamine, serotonin, GABA and glutamate) (for review, see [27]). Furthermore, changes in DNAm have also been described in genes involved in neuroplasticity regulation, such as brain-derived neurotrophic factor (BDNF) and mediators of the neuroendocrine response to stress (glucocorticoid receptors and neuropeptides) [76]. Mediators of the inflammatory response, such as cytokines, are also known to have their expression regulated by DNAm in response to stress and psychiatric disorders [27,77]. Such changes could compromise the neurochemical milieu and synaptic homeostasis required for promoting stress adaptation, thereby hindering resilience and increasing the vulnerability to psychiatric disorders by different mechanisms [78,79].

DNAm is also influenced by genetic variation. Distinctly methylated genes seem to impact DNAm on phenotype differences, such as susceptibility to certain diseases and pathogens and response to drugs and environmental agents [80]. Many genome-wide association studies have provided a growing list of genetic variations associated with psychiatric phenotypes and have clarified the shared and unique components of mental illness [81,82,83]. A genome-wide meta-analysis study analyzing eight psychiatric disorders has found 146 risk loci, of which 109 are associated with at least two psychiatric disorders, confirming the effect of genetic risk variants and highlighting the close genetic relationship between some diseases, such as schizophrenia and bipolar disorder [84]. Genetic variants in DNMTs are critical in defining the threshold for environmental factors toward susceptibility to psychiatric diseases [28]. Therefore, a substantial fraction of the epigenome is controlled by the DNA sequence, and the genetic–epigenetic paradigm is important to understand how genes and life adversities outline individuals in states of vulnerability or resilience [85].

Interestingly, drugs used to treat psychiatric disorders, such as antidepressants, mood stabilizers and antipsychotics, can correct some, but not all, DNAm changes observed in stressed animals and patients [75,86,87], thus suggesting that this mechanism can contribute to their therapeutic effects. In line with that view, preclinical evidence has demonstrated that drugs that directly target DNMTs with resulting inhibition of their catalytic activity, such as decitabine or RG108, promote stress-coping behavior in different animal models of psychiatric disorders: the forced swim test [88,89], the learned helplessness [64] and the social defeat stress [90,91]. Therefore, DNAm can be explored as a possible pharmacological target to correct aberrant methylation patterns associated with increased vulnerability to disease, including stress-induced psychiatric disorders [87,90,92]. Drugs with potential ability to regulate the activity and/or the expression of DNMTs and TETs become, thus, valuable therapeutic tools for further research.

### 3.2. CBD Effects on DNAm: In Vivo Evidence

Evidence describing CBD effects on DNAm is still scarce. When searching on PubMed using the search string [(*cannabidiol*) AND (*DNA methylation*) (ALL FIELDS)], only 17 papers were retrieved (30 August 2022). Of those, two are not dealing with CBD effects on DNAm [93,94]; one used a mixture of polyphenols, including CBD, making it difficult to draw any conclusion regarding CBD effects [95]; and four are narrative reviews [96,97,98,99], as summarized in Table 1.

The first piece of evidence describing the possible effects of CBD on DNAm aimed at clarifying the epigenetic regulation of keratinocyte differentiation by phytocannabinoids, including CBD [100]. This investigation using human kHaCaT cells revealed that CBD (0.1–1.0 µM, 5 days) increased global DNAm, DNMT1 expression level and DNMT activity, and decreased gene expression involved in keratinocyte differentiation [100]. Interestingly, CBD effects on DNAm and gene expression were mimicked by AEA and blocked by a selective CB1 antagonist, suggesting an indirect mechanism rather than a direct regulation of DNMT activity by CBD [100].

In another study, the neuroprotective effects of CBD against mitochondrial dysfunction induced by neonatal iron overload were investigated in the hippocampus of adult Wistar rats [101]. Iron exposure reduced 5mC and 5hmC levels in mitochondrial DNA in the hippocampus, an effect attenuated by CBD treatment (10 mg/kg, 14 days) in adulthood [101]. Surprisingly, there were no corresponding changes in the expression of mitochondrial ferritin, but the lack of analysis of other mitochondrial genes makes it difficult to draw any conclusions about possible epigenetic mechanisms of CBD in this context. Nevertheless, given the importance of mitochondrial genes and metabolism for determining susceptibility to stress-induced psychiatric disorders [110,111,112], this mechanism could be relevant for the therapeutic effects of CBD in such conditions.

The epigenetic mechanisms of CBD were also investigated in neurodevelopmental models associated with the development of schizophrenia. Chronic postnatal CBD treatment (30 mg/kg/day; 10 days) rescued the decreased sociability and recognition memory deficit induced by prenatal exposure to the antimitotic agent methylazoxymethanol acetate (MAM), which is a model to study the negative symptoms and cognitive deficits associated with schizophrenia [25,97,98,102]. Moreover, CBD attenuated the decreases in DNAm and increases in mRNA expression of CB1 receptors in the prefrontal cortex of MAM-treated animals, implicating epigenetic mechanisms in the antipsychotic properties of CBD [102]. In another study, the perinatal exposure to THC also induced neurodevelopmental deficits associated with a schizophrenia-like phenotype in rats. This observation was made along with decreased DNAm and increased expression of D2 receptors in the prefrontal cortex, effects that were attenuated by peripubertal treatment with CBD (30 mg/kg/day, 10 days) [105]. Interestingly, decreased DNAm in parallel with increased expression of the D2 receptor gene was found in blood cells of schizophrenic patients [99,105], and all known antipsychotics block D2-mediated signaling in the mesolimbic pathway. Furthermore, differential DNAm at multiple loci across the genome are associated with psychosis and schizophrenia [113]. Therefore, it is possible to speculate that, at least in part, the antipsychotic effects of CBD might involve the regulation of altered gene expression in the mesolimbic pathways due to changes in DNAm patterns of genes relevant for this disorder.

Corroborating those findings, acute CBD administration (60 or 30 mg/kg) attenuated the dysfunctions in the sensorimotor gating induced by psychomimetic drugs in adult Wistar rats associated with changes in global DNAm in two brain regions [107]. However, CBD effects in the corresponding DNAm changes were rather complex, both increasing or decreasing global DNAm depending on the brain region analyzed (prefrontal cortex vs. ventral striatum) and the drug used as the psychomimetic (amphetamine or MK-801) [101]. Altogether, these studies suggest that the antipsychotic effect of CBD might involve dynamic and tissue-specific regulation of DNAm in schizophrenia, but the methods used in such studies do not allow for further conclusions regarding the precise mechanisms involved in CBD effects. The dual regulation of DNAm described in the study of Pedrazzi and colleagues [107] suggests indirect mechanisms due to CBD affecting different neurotransmitter systems involved in schizophrenia (e.g., D2 and 5-HT1A), as it seems to be the case for antipsychotic drugs [114]. Therefore, future studies should consider the analysis of DNAm of individual genes through more advanced techniques, such as Whole-Genome Bisulfite Sequencing (WGBS), to evaluate CBD effects compared with known antipsychotic drugs.

The effects of CBD on stress-induced DNAm have also been investigated to evaluate if its antidepressant properties could be associated with rapid changes in gene expression in brain regions relevant for depression neurobiology. In adult Wistar rats submitted to the forced swimming test, an animal model predictive of antidepressant effects [115], acute CBD administration (10 mg/kg) attenuated the immobility time in the test and reversed the decreased and increased DNAm in the prefrontal cortex and hippocampus, respectively, induced by stress [103]. Moreover, the combination of subeffective doses of CBD (7 mg/kg) with subeffective doses of two chemically unrelated inhibitors of DNAm (RG108 and 5-aza-2-deoxycytidine, 5-azaD) promoted a significant antidepressant effect but without corresponding synergistic effects in DNAm [103]. Surprisingly, the DNMT activity was affected by CBD treatment only in the prefrontal cortex but not in the hippocampus, highlighting again tissue-specific effects of the compound on DNAm patterns across the genome Although the interpretation of these findings is limited by the measurement of global DNAm and DNMT activity, it is possible to speculate the involvement of indirect mechanisms, given the dual nature of the changes. However, RG108 and 5-azaD promoted the same dual changes as CBD, indicating the involvement of more complex mechanisms. Previous evidence suggested that the mammalian DNMTs can also act as Ca2+ ion- and redox-state-dependent active DNA demethylases [116], which makes it possible that drug binding to the enzyme (by CBD, RG108 or 5-azaD) could either increase or decrease activity-induced changes in DNAm, as observed by Sales and colleagues [103].

It is worth noting that acute treatment with conventional monoaminergic drugs promoted similar effects as CBD in swim-induced changes in DNAm and also presented synergistic effects when combined with RG108 or 5-azaD [88]. Furthermore, chronic—but not acute—treatment with monoaminergic antidepressants attenuated the increase in DNAm and DNMT3 levels in the prefrontal cortex of rats exposed to the learned helplessness model of depression, thus implicating this epigenetic mechanism in the delayed antidepressant effect [117]. Interestingly, both CBD (30 mg/kg) [118] and DNAm inhibitors (RG108 and 5-azaD) [64] induced fast and sustained antidepressant effects in rats exposed to different models of depression, including the learned helplessness, with the involvement of increased signaling by BDNF and its receptor, TrkB, in the prefrontal cortex. Although increased DNAm and decreased gene expression levels of BDNF and TrkB are described in the brains of stressed animals and depressed subjects [87,119], it is not known if CBD can reverse such changes.

DNAm plays an essential role in the neurobiology of depression. It has been associated with modifications in genes such as the serotonin transporter (*SLC6A4* or *5-HTT*), BDNF, glucocorticoid receptor (*NR3C1* or *GR*), mineralocorticoid receptor (*NR3C2* or *MR*), FK506-binding protein 5 (*FKBP5*) and corticotropin-releasing hormone receptor 1 (*CRHR1*) [27,75]. Antidepressant drugs can modulate DNAm in the promoter region of genes related to neuroplasticity and mood regulation [87]. Methylomic and transcriptomic studies can provide important additional information to indicate if changes in DNAm are paralleled by corresponding transcriptional changes in the genes involved with depression neurobiology and/or the antidepressant effect.

In fact, two recent studies investigated genome-wide changes in brain DNAm patterns induced by CBD, but none used animal models of psychiatric disorders [104,106]. In one study, the authors investigated the neurodevelopmental effects of CBD exposure (from before breeding to lactation) on behavior and genome-wide methylation profile in F0 and F1. Developmental exposure to CBD results in sex-specific increases in anxiety and memory performance in F1 with hyper- and hypomethylation in both directly and developmentally exposed animals (predominantly hypomethylation in F1) [106]. Functional enrichment analysis revealed an over-representation of genes involved in neurogenesis and neuron morphology, while top disease terms pointed to autism spectrum disorder, schizophrenia and intellectual disability [106]. In another study with naive adult female wild-type mice that received CBD (20 mg/kg/day, 14 days), a small skew toward global hypomethylation was observed in the hippocampus, including hypomethylation of the de novo methyltransferase DNMT3a and 3.323 differentially methylated loci enriched for genes involved in neuronal function and synaptic organization [104]. Disease ontology enrichment revealed an over-representation of differentially methylated loci in gene sets associated with autism spectrum disorder and schizophrenia [104]. The results of both studies confirm CBD effects on DNAm changes, which vary depending on sex, age and duration of exposure. Since both studies employed naive animals, genome-wide methylation analysis of CBD effects in animals exposed to different models of psychiatric disorders is required to decipher CBD effects in DNAm, the mechanisms involved and their functional consequences.

The current evidence does not provide clear information about the mechanisms involved in CBD’s effects on DNAm. There are, however, some possibilities to be considered:

(a) CBD could regulate DNAm by indirectly changing the availability of neurotransmitters, such as eCB and glutamate. As previously mentioned herein, CBD can increase AEA availability [14], and AEA can induce DNMT activity in a CB1-dependent manner involving p38 MAPK signaling in differentiated keratinocytes [120]. CBD can also regulate glutamate levels by blocking its reuptake or indirectly by increasing eCB levels with subsequent CB1 activation, thereby inhibiting neurotransmitter release [121]. Activation of NMDA receptors can regulate DNMT3 activity/expression levels through a CREB-dependent mechanism [122].
(b) CBD could directly target enzymes involved in methylation and demethylation of the DNA, such as DNMTs and TETs, respectively. Currently, there is no evidence that CBD could bind and/or regulate the activity of DNMTs. However, a recent publication indicates that CBD and other related cannabinoids exhibit potent inhibitory activities towards the TET1 protein in vitro, most likely due to interaction with amino acid residues in the active center of the enzyme, according to an in silico molecular docking approach [108].

### 3.3. CBD Effects on DNAm: In Silico Evidence

As mentioned above, evidence regarding the potential binding affinities of CBD for enzymes involved in DNAm could provide important information regarding the mechanism of action of CBD on transcriptional regulation and neuroplasticity. A recent docking study using a homology model of TET1 based on the alignment to a solved crystal structure of the human TET2 enzyme suggests that CBD interacts with amino acid residues in the active center of the enzyme essential for its inhibition [108]. Although further in vivo experiments are necessary to confirm this hypothesis, it poses an interesting new mechanism of action for CBD. Considering that both overexpression of TET1 and its catalytically inactive mutant affected gene expression and memory formation in similar ways, including the expression of neuroplasticity-related genes [72], it is difficult to speculate about the impact that TET1 inhibition by CBD would have on the control of gene expression. One promising study, however, identified that the selective deletion of TET1 in the nucleus accumbens promotes antidepressant-like effects in mice submitted to social defeat stress [123]. It is, thus, possible that some of CBD effects involve DNAm changes due to the regulation of TET1 activity in the nucleus accumbens. This hypothesis requires further investigation.

**Figure 5:**
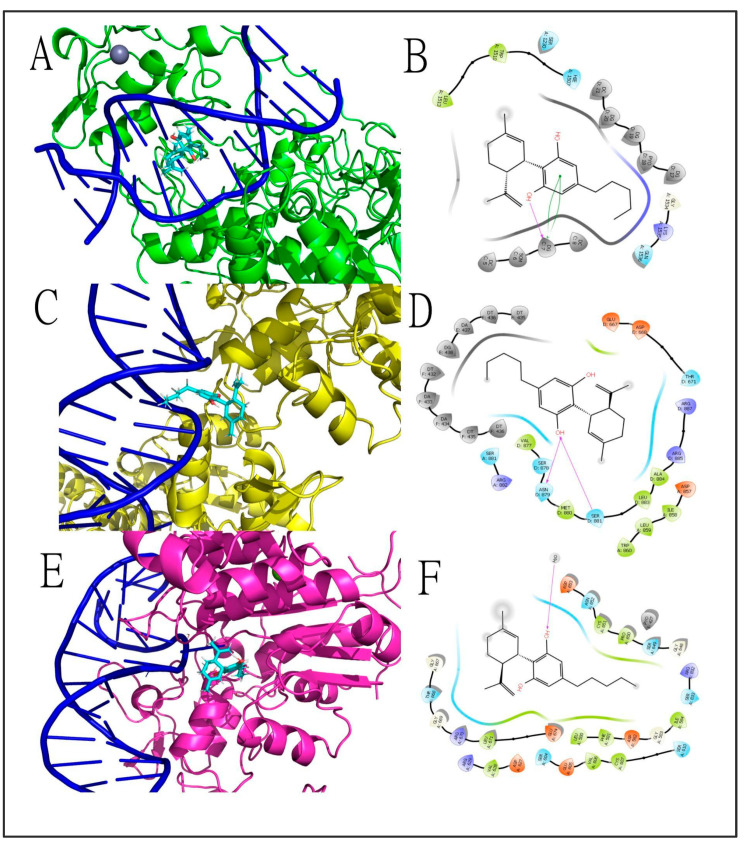
The 3D ((**A**,**C**,**E**) in left panels) and 2D ((**B**,**D**,**F**) in right panels) intermolecular interactions among cannabidiol and DNMT targets. Cannabidiol docked in (**A**,**B**) DNMT1 with a docking score of −6.230 kcal mol^−1^ and LE: −0.271 for the best conformer and made hydrogen bonds (magenta arrow lines) and π–π stacking interactions (green curved lines) with nucleotides of DNA double-helix; in (**C**,**D**), DNMT3a (docking score −5.684 kcal mol^−1^, LE: −0.247) made hydrogen bonds (magenta arrow lines) with amino acid residues of the binding site of the protein; and in (**E**,**F**) DNMT3b (docking score −2.897 kcal mol^−1^, LE: −0.126) made a single hydrogen bond (magenta arrow lines) with a solvation water molecule around the protein–DNA complex. In all three protein targets, CBD made a net of hydrophobic and polar interactions with amino acid residues, represented by green and blue drop-shapes, respectively. Cannabidiol was represented in Licorice model with carbon atoms represented in cyan, oxygens in red and hydrogens in white. Proteins and DNA helix were represented in Newcartoon model with carbon atoms of DNMT1 represented in green, DNMT3a in yellow and DNMT3b in deep salmon. DNA double helix was represented in dark blue. The 2D diagrams were built at the Maestro, Schrodinger suite, and 3D diagrams were generated with the Pymol software. Abbreviations: CBD—cannabidiol; DNMTs—DNA methyltransferases; LE—ligand efficiency.

To further explore the possibility that CBD would target other components of the epigenetic machinery, we performed an in silico evaluation to test CBD’s affinity to the different DNMTs (DNMT1, DNMT3a and DNMT3b) through molecular docking assays. To do that, we used crystallographic structures of the corresponding human proteins retrieved from Protein Data Bank (PDB) [41], with their respective PDB IDs: DNA Methyltransferase 1 (DNMT1)—6 × 9J [124]; DNA Methyltransferase 3 α (DNMT3a)—6BRR [125]; and DNA Methyltransferase 3 β (DNMT3b)—6U8P [126]. Only structures with co-crystallized ligands and with resolution levels (in Angstroms, Å) equal to or better than 3.0 Å were used. Molecular docking calculations were performed using Glide—Schrödinger, considering the ligands flexible and the protein rigid. CBD structure was retrieved from the PubChem repository (PUBCHEM ID 13956-29-1) and it was docked into DNMTs using the Maestro Glide software in extra-precision (XP) mode [127]. The complete methodological approach can be found in the Supplementary Material provided, and the results are represented in Figure 5 and Table S1.

Briefly, the findings of the docking study indicate that CBD has a similar docking score with a superior ligand efficiency (LE) score to DNMT1 when compared to the control co-crystallized inhibitor, GSK3685032. Interestingly, CBD interacts in a different site in the DNMT1 binding pocket, making more interactions with the nucleotides of the open-frame DNA double-helix than GSK3685032, which interacts more directly with some amino acid residues. CBD intermolecular interactions with DNMT3a and DNMT3b were few and the calculated docking scores were inferior to those of the co-crystallized ligand S-adenosyl-L-homocysteine (SAH). As depicted in Figure 5, CBD interacts in a different and possibly complementary binding pocket of DNMT1, probably contributing to a steric impediment of this enzyme activity in the DNA double helix. This is likely to result in direct inhibition of the enzyme by CBD, which could explain the already described inhibitory effects of CBD upon DNAm in stressed animals and synergistic effects when administered concomitantly with DNAm inhibitors [103].

## 4. Conclusions

CBD has a strong therapeutic potential for the treatment of stress-related psychiatric disorders, such as depression, anxiety and schizophrenia [10,18]. Despite the many pharmacological targets already disclosed for CBD, the precise contribution of each of them to CBD effects remains poorly understood. The evidence reviewed herein suggests an important contribution of the regulation of gene expression by direct and/or indirect targeting of the enzymes that catalyze DNA methylation/demethylation, with possible subsequent effects for the transcription of genes associated with the neurobiology of psychiatric disorders. The interference with DNAm by CBD indicates that it may have transgeneration and neurodevelopmental effects, which may also be associated with pathophysiological mechanisms and susceptibility to disease [98,104]. Future studies should better characterize CBD effects on DNAm using in vitro and in vivo approaches to describe epigenetic changes in animals of both sexes, at different stages of development, across tissues, under stressful and non-stressful conditions. Finally, since there are no studies to date that analyzed CBD effects on DNA methylome in humans, the analysis of DNAm changes in the blood as well as postmortem brain tissues of patients exposed to treatment with CBD could also reveal important epigenetic changes induced by the drug and its potential implications in psychiatry.

## Figures and Tables

**Table 1:** Published evidence about cannabidiol effects on DNAm (search: cannabidiol and DNA methylation, PubMed, 30 August 2022).

Article Categories	Cell or Tissue Type	Type of Study	CBD Effects Related Psychiatric Disorder	Gene	DNAm Measure	Main Findings	Assessment	Reference
Comparative study	Keratinocytes human HaCaT cells	In vitro	N.A.	Keratin 10 and global DNAm	DNAm-specific primed PCR Methyl-accepting assay with CpG methylase SssI	CBD (0.5 µM) increased DNAm of keratin 10 gene CBD (0.5 µM) increased global DNAm by selectively enhancing DNMT expression	First evidence that CBD could target DNAm	[100]
Research study	Hippocampal mitochondria	Animal model (male Wistar rats)	Neurodegenerative	N.A.	Methylated DNA quantification ELISA kit	CBD (10 mg/kg/day, i.p.; 14 consecutive days) attenuated iron-induced decrease in global DNAm	First evidence that CBD restores hippocampal DNAm of mitochondrial mtDNA	[101]
Research study	Prefrontal cortex	Animal model (male Sprague-Dawley rats)	Schizophrenia	CNR1	Pyrosequencing of bisulfite converted DNA	CBD (30 mg/kg/day, i.p.; 20 days) reduced DNAm in the CNR1 promoter	First evidence regarding the involvement of DNAm in the antipsychotic properties of CBD	[102]
Review	N.A.	N.A.	Fragile X Syndrome	N.A.	N.A.	N.A.	None about CBD and DNAm	[99]
Review	N.A.	N.A.	N.A.	N.A.	N.A.	N.A.	Brief review of published articles	[98]
Research study	Prefrontal cortex and hippocampus	Animal model (male Swiss mice)	Depression	Global DNAm	DNAm ELISA kit	CBD (10 mg/kg, i.p.) attenuated the DNAm changes induced by stress (increasing DNAm in the prefrontal cortex and the decreasing DNAm in the hippocampus)	First evidence regarding the involvement of DNAm in the antidepressant-like action of CBD	[103]
Research study	Hippocampus	Animal model (male *Agouti viable yellow- A^vy^* mice)	Autism, schizophrenia	N.A.	Genome-wide DNAm (reduced-representation bisulfite sequencing)	3323 genes’ differentially methylated loci were found in CBD-exposed animals (20 mg/kg/day, p.o.; 14 days)	CBD modifies DNAm in genes relevant for psychiatric diseases	[104]
Research study	Prefrontal cortex and PBMCs	Animal model (male Sprague-Dawley)	Schizophrenia	DRD2 dopamine D2 receptor	Pyrosequencing	DRD2 DNAm (CpG site 1) was reduced in the PBMCs of schizophrenic subjects CBD (30 mg/kg/day, i.p.; 21 consecutive days) attenuated the DRD2 DNAm reduction in the prefrontal cortex of rats exposed to the THC	Peripubertal CBD treatment reverted DNAm modulation of DRD2 in rats	[105]
Comparative study	Cortex and hippocampus	Animal model (female *A^vy^* mice)	Neurodevelopmental disorders, epilepsy and others	N.A.	Genome-wide DNAm (reduced-representation bisulfite sequencing)	CBD (20 mg/kg/day, i.p.; 14 consecutive days)	First evidence that developmental CBD exposure modified DNAm	[106]
Review	N.A.	N.A.	Mood disorders and anxiety	N.A.	N.A.	N.A.	Showed evidence that therapeutic effects of CBD could involve DNAm	[97]
Research study	Canine monocyte-macrophage (DH82) and epidermal keratinocytes cells	In vitro	Canine atopic dermatitis	Ccl2, ccl17 and tslp, il31ra	Bisulfite-treated DNAm pyrosequencing	The nutraceutical mixture induced a significant downregulation of many genes in immune cells, along with increased DNAm	CBD effects were not investigated isolated, but only as part of the nutraceutical treatment (mixture containing polyphenols and cannabinoids), making it difficult to assess CBD effects	[95]
Research study	Ventral striatum and prefrontal cortex	Animal model (male Swiss mice)	Schizophrenia	Global DNAm	DNAm ELISA kit	CBD (30 and 60 mg/kg, i.p.) prevented the amphetamine-induced DNAm increase in the ventral striatum	First evidence that CBD has sustained antipsychotic-like action, suggesting the involvement of DNAm in these effects	[107]
Review	N.A.	N.A.	Mood disorders and schizophrenia	N.A.	N.A.	N.A.	None about CBD and DNAm	[96]
Research study	N.A.	Silico molecular docking	N.A.	N.A.	N.A.	Cannabinoids, including CBD, inhibited the activity of TET1 protein	First in silico evidence that CBD can regulate DNAm through direct interaction with TET1 Did not investigate CBD effects on DNAm	[108]
Research study	Leaf-originated explant	In vitro	N.A.	N.A.	Methylation-sensitive amplification polymorphism	The plasma treatment induced differential DNA methylome	Did not investigate CBD effects on DNAm	[94]
Research study	Callus cells of Cannabis indica	In vitro	N.A.	N.A.	Methylation-sensitive amplification polymorphism	Simulated microgravity-triggered changes in the DNAm profile	Did not investigate CBD effects on DNAm	[93]
Review	N.A.	N.A.	Depression	N.A.	N.A.	N.A.	Showed that multiple genes related with depression are differentially methylated upon exposure to the cannabis or cannabis-derived compounds, including CBD	[109]

Note: DNAm—DNA methylation; N.A.—not applicable; PCR—polymerase chain reaction; CpG—cytosine phosphate guanine; CBD—cannabidiol; DNMT—DNA methyltransferase; mtDNA—mitochondrial DNA; CNR1—cannabinoid receptor 1; ELISA—enzyme-linked immunosorbent assay; DRD2—dopamine receptor D2; TET—ten-eleven translocation.

## Supplementary Materials

The following supporting information can be downloaded at: https://www.mdpi.com/article/10.3390/genes13112165/s1, Table S1: Docking results of cannabidiol against DNA methyltransferases (DNMTs).
