# Supplementary material for: Regulation of DNA Methylation by Cannabidiol and Its Implications for Psychiatry: New Insights from In Vivo and In Silico Models
Source: Genes (Basel). 2022 Nov 20;13(11):2165. doi: 10.3390/genes13112165 (PMC9690868; doi:10.3390/genes13112165)
Supplement: Supplementary file 1 [file genes-13-02165-s001.zip › genes-1991411-supplementary.pdf]

Supplementary Material

In Silico Evaluation of Cannabidiol Affinity to DNA Methyltransferases through Molecular Docking Assays

Molecular docking calculations were performed using Glide - Schrödinger, considering the ligands flexible and the protein rigid. The structure of the cannabidiol (CBD) was retrieved from the PubChem repository (PUBCHEM ID 13956-29-1). The CBD ligand was docked into DNMTs using the Maestro Glide software in extra-precision (XP) mode [121]. The protein structures were prepared using the Protein Preparation Wizard tool [122], adding the hydrogen atoms and minimizing the energy, using the OPLS-2005 force field [123]. The CBD ligand was prepared through the LigPrep tool, correcting protonation, according to Epik, generating conformers and performing energy minimization [122,124]. The protein grid coordinates for all DNMTs were centered around the co-crystallized ligands in their active pocket sites with a radius distance of 10 Å. For DNMT1, the grid was centered around the ligand GSK3685032, a potent first-in-class DNMT1-selective inhibitor [118]. For DNMT3A and DNMT3B, the grid was centered around the ligand s-adenosyl-L-homocysteine (SAH), an endogenous inhibitor [119,120]. For the docking calculations, these co-crystalized ligands were excluded from the grid to avoid possible bias interactions with CBD. To allow a possible comparison with CBD docking scores, the co-crystallized ligands were extracted from the protein structure as new entries, prepared through a similar protocol with LigPrep and submitted to docking calculations using the Maestro Glide software in the same extra-precision (XP) mode. Docking scores were expressed as kcal mol−1 as a parameter of potential ligand affinity. More recently, ligand efficiency (LE), defined as affinity or docking scores divided by the number of heavy atoms in a molecule, was introduced as a useful parameter to guide the selection of promising drug candidates [125]. Both parameters were represented for CBD and co-crystallized ligands in the selected protein targets. The non-covalent interactions between CBD and the proteins were represented in 2D-diagrams generated by Maestro suite. The The PyMOL Molecular Graphics System, Schrödinger, LLC was used for the visual inspection of the docking poses and to render the 3D molecular images.

Table S1. Docking results of cannabidiol against DNA methyltransferases (DNMTs).

Protein	PDB ID	Drug	Docking Score (kcal mol−1)	Ligand Efficiency
DNMT1	6X9J	Cannabidiol	-6.230	-0.271
		GSK3685032	-7.689	-0.240
DNMT3A	6BRR	Cannabidiol	-5.684	-0.247
		S-adenosyl-L-homocysteine (SAH)	-8.685	-0.334
DNMT3B	6U8P	Cannabidiol	-2.897	-0.126
		S-adenosyl-L-homocysteine (SAH)	-10.564	-0.406

Abbreviations: CBD – cannabidiol; DNMTs - DNA methyltransferases; PDB ID: Protein Data Bank Identification.
